# The Sweet and Salty Dietary Face of Hypertension and Cardiovascular Disease in Lebanon

**DOI:** 10.3389/fphys.2021.802132

**Published:** 2022-01-25

**Authors:** Mohammad M. Labban, Maha M. Itani, Dina Maaliki, Zeina Radwan, Lara Nasreddine, Hana A. Itani

**Affiliations:** ^1^Faculty of Medicine, University of Balamand, Beirut, Lebanon; ^2^Department of Pharmacology and Toxicology, Faculty of Medicine, American University of Beirut, Beirut, Lebanon; ^3^Department of Anatomy, Cell Biology and Physiological Sciences, Faculty of Medicine, American University of Beirut, Beirut, Lebanon; ^4^Vascular Medicine Program, American University of Beirut Medical Center, Beirut, Lebanon; ^5^Department of Nutrition and Food Sciences, Faculty of Agricultural and Food Sciences, American University of Beirut, Beirut, Lebanon; ^6^Adjunct Clinical Pharmacology, Vanderbilt University Medical Center, Nashville, TN, United States

**Keywords:** lifestyle, diet, salt, fructose, hypertension, immunity

## Abstract

According to the World Health Organization (WHO), an estimated 1.28 billion adults aged 30–79 years worldwide have hypertension; and every year, hypertension takes 7.6 million lives. High intakes of salt and sugar (mainly fructose from added sugars) have been linked to the etiology of hypertension, and this may be particularly true for countries undergoing the nutrition transition, such as Lebanon. Salt-induced hypertension and fructose-induced hypertension are manifested in different mechanisms, including Inflammation, aldosterone-mineralocorticoid receptor pathway, aldosterone independent mineralocorticoid receptor pathway, renin-angiotensin system (RAS), sympathetic nervous system (SNS) activity, and genetic mechanisms. This review describes the evolution of hypertension and cardiovascular diseases (CVDs) in Lebanon and aims to elucidate potential mechanisms where salt and fructose work together to induce hypertension. These mechanisms increase salt absorption, decrease salt excretion, induce endogenous fructose production, activate fructose-insulin-salt interaction, and trigger oxidative stress, thus leading to hypertension. The review also provides an up-to-date appraisal of current intake levels of salt and fructose in Lebanon and their main food contributors. It identifies ongoing salt and sugar intake reduction strategies in Lebanon while acknowledging the country’s limited scope of regulation and legislation. Finally, the review concludes with proposed public health strategies and suggestions for future research, which can reduce the intake levels of salt and fructose levels and contribute to curbing the CVD epidemic in the country.

## Introduction

Cardiovascular diseases (CVDs) are the leading cause of death globally, accounting for 17.9 million deaths annually (World Health Organization, 2021). The Eastern Mediterranean Region (EMR) is no exception, with CVDs accounting for approximately 50% of overall mortality in the region (Al Jawaldeh et al., 2018). In Lebanon, a Middle Eastern country of the Mediterranean basin, CVDs were estimated to contribute to approximately 47% of mortality in individuals above the age of 50 (World Health Organization, 2019). The major risk factor for CVD morbidity and mortality is hypertension (HTN), a cardiovascular condition that may result from suboptimal diets and food consumption practices. Like other countries of the Middle East, Lebanon is witnessing the nutrition transition, accompanied by its characteristic shifts in diet and lifestyle (Sibai et al., 2010). The hallmark of the nutrition transition is a shift away from the traditional Lebanese dietary pattern, a variant of the Mediterranean diet, toward a more westernized dietary pattern, characterized by increased intakes of energy-dense processed foods that are high in fat, salt, and added sugar. High intakes of salt and added or free sugar have been linked to the etiology of HTN in different cross-sectional and longitudinal studies (Stolarz-Skrzypek, 2011; Genovesi et al., 2021), hence potentially contributing to the increasing burden of CVDs.

The American Heart Association (AHA) has, in fact, been increasingly emphasizing the promotion and support of healthful behavior changes, including dietary practices, for the prevention and treatment of CVDs and their metabolic risk factors (Angell et al., 2020). Dietary strategies that reduce CVDs include adopting diets that are lower in salt and sugar (Sikand and Severson, 2020). The World Health Organization has specifically recommended limiting free sugar intake to less than 10 percent of calories per day (World Health Organization, 2015), with another conditional recommendation further limiting its intake to less than 5% of energy. Sources of free sugar mainly include table sugar and high-fructose corn syrup (HFCS) and naturally occurring sugar in fruit juices and syrups (Malik and Hu, 2015). Table sugar or sucrose is a disaccharide composed of one molecule of fructose and one glucose molecule, while HFCS contains up to 55% fructose (Malik and Hu, 2015). HFCS is widely used in commercial food like cereals, soft drinks, sports drinks, and others. Although fructose is a monosaccharide naturally present in fruits and honey and plays an essential role in glycolysis and gluconeogenesis (Wolf, 1995), its total consumption has been found to increase as a reflection of its use in processed foods. For instance, fructose intake has increased in the United States by 33% between the mid-1970s and the early 2000s, with the highest consumption observed in young adults between 19 and 22 years old mainly due to increased intake of sweetened beverages (Marriott et al., 2009). These facts raise a public health concern since studies showed that high fructose consumption is associated with insulin resistance, fatty liver disease, obesity, and hypertension (Stanhope et al., 2009; Jalal et al., 2010; Tappy and Lê, 2010). While several observational analyses have disputed the nature and magnitude of the association between sodium and vascular outcomes (Alderman, 2014; Campbell et al., 2014; Mente et al., 2014; O’Donnell et al., 2014), the available evidence supports lowering the population’s sodium consumption as an effective public health strategy (Trieu et al., 2015; Milajerdi et al., 2019). Dietary sodium intake is the sum of small amounts of sodium present in food. Sources of dietary sodium can be “discretionary” (from salt added during the preparation of food in the kitchen or at the table) or “non-discretionary” (from the sodium that is found naturally in food or added during industrial food transformation). The “non-discretionary” sources of sodium are mainly in the form sodium chloride, with ∼0.10 g being in the form of monosodium glutamate (MSG), sodium bicarbonate (baking soda), sodium nitrite, and sodium benzoate. Most developed countries’ diets are high in sodium, with approximately 70% of sodium in the diet is in processed food including breads, salted meats, canned goods, cereals, pastries, and food preparation (fast-food and sit-down restaurants) (Anderson et al., 2010; Centers for Disease Control and Prevention, 2012). For example, 1.5–2 g of salt per 100 g may be found in bread, while the sodium levels in meat and cheese is extremely high (up to 2.5 g/100 g). In a recent systematic review of prospective studies, there was evidence of a direct association between urinary excretion of sodium and the risk of CVD mortality. This association was more significant at sodium intakes exceeding 2.4 g/day (Milajerdi et al., 2019). The United Kingdom’s sodium reduction strategy provides further support with regards to the feasibility and impact of salt/sodium reduction interventions, showing a 15% decrease in the population’s sodium intake between 2003 and 2011 (Sadler et al., 2011), which was accompanied by a significant reduction in the population’s average blood pressure by 3/1.4 mm Hg (He et al., 2014). At the 66th World Health Assembly, which took place in 2013, the WHO Member States committed to the global target of a 30% relative reduction in mean population intake of sodium by 2025, against a 2010 baseline (Target 4) (World Health Organization, 2014). It is also expected that efforts to meet the global target on sodium reduction will contribute toward achieving the Sustainable Development Goals (SDGs), including Target 3.4 of reducing premature NCD-related mortality by at least a third (United Nations Statistics Division, 2016).

In a country where the intake of both sugar and salt increases with a concomitant increase in the burden of hypertension and CVDs, this review aims to describe the evolution of hypertension and CVDs in Lebanon and elucidate the potential mechanisms of fructose-induced hypertension, salt-induced hypertension, and the effect of salt-fructose combinations on blood pressure regulation. The review will also provide an up-to-date appraisal of current intake levels of salt and fructose in Lebanon and their main food contributors. It shall conclude with proposed public health strategies, which can reduce the intake levels of salt and fructose levels and contribute to curbing the CVD epidemic in the country.

## Hypertension and Cardiovascular Diseases Evolution in Lebanon

Hypertension is a major cause of CVD and its consequences worldwide, yet its prevalence among countries varies. Three-quarters of the world’s hypertensive population lives in low- and middle-income countries, with limited health resources, little awareness of hypertension, and limited access to health treatment (Mills et al., 2020). Lebanon, a small upper-middle-income Arab country, was reported to have a prevalence of hypertension. Based on recent studies in Lebanon, hypertension affects one-third of the population, with another 30% being pre-hypertensive (Matar et al., 2015). More concerning is the reported secular trend, which has shown a threefold increase in the prevalence of hypertension in Lebanon over the last decade (Tohme et al., 2005). Most recently, epidemiological data assessing the prevalence, awareness rate, and control rate of hypertension in Lebanon reported a prevalence of 36.4%, with an awareness rate of 65.4% and a controlled rate of 61% (Noubani et al., 2018). The authors concluded that national public health interventions are urgently needed to combat the rising prevalence of hypertension, accomplish primary prevention, and better control the disease. Since 1995, CVDs have been the leading cause of death; CVDs accounted for 33% of deaths worldwide (Institute for Health Metrics and Evaluation and Global Burden of Disease Collaborative Network, 2017). CVD is the major cause of death and hospitalization reported by the Lebanese Ministry of Public Health, as it is in the rest of the world (Isma’eel et al., 2021). Although hypertension is a risk factor for CVD, in fact, systolic blood pressure (SBP) is more frequent than diastolic blood pressure (DBP) and is more closely linked to cardiovascular problems (Lloyd-Jones et al., 2000). A study reported that 24.9% of patients with uncontrolled DBP were associated with CVD, while 43.1% of patients with uncontrolled SBP were associated with CVD (Zeidan et al., 2016).

Compared to other countries globally, the incidence rate of hypertension and CVDs among the Lebanese population falls in the middle range. Being one of the Arab countries, when compared to other regions of the world, such as Sub-Saharan Africa and the United States, the Arab world had a higher crude prevalence of hypertension with 29.5% vs. 28% prevalence in the United States and 27.5% prevalence in Sub-Saharan Africa (Tailakh et al., 2014).

## Mechanisms Linking High Sodium and High Fructose Intakes to HTN

### High-Sodium Induced Hypertension

Salt-sensitive hypertension is an essential component of hypertension affecting approximately 50% of hypertensive patients, leading to a threefold increase in the risk of cardiovascular events compared to salt-resistant hypertensive patients. Excess dietary sodium (Na^+^) is a significant risk factor for hypertension and CVDs. Since table salt is the most prevalent source of dietary sodium, the World Health Organization (WHO) recommends that adults reduce sodium intake to less than 5 g of salt/day (2,000 g of sodium/day). A high salt diet induces hypertension through various mechanisms.

#### Inflammation

A correlation between salt-sensitive hypertension and renal inflammation has been the center of findings in research. Tubulointerstitial inflammation has been found to impair the excretion of salt from the kidneys. This leads to increased salt sensitivity, which thus can cause hypertension. It was also found that inflammation-suppressing drugs can prevent hypertension from developing (Herrera et al., 2006; Rodriguez-Iturbe and Johnson, 2010). In addition, it has been widely observed that inflammation and lymphocyte (CD3, CD4, CD8) and macrophage (CD-68) accumulation in kidneys and arteries are widespread among hypertensive patients (Franco et al., 2013; Rodríguez-Iturbe et al., 2014). Others found that kidney injury and thus inflammation causes enhanced sodium reabsorption, leading to hypertension in mice (Lombardi et al., 1999).

#### Aldosterone-Mineralocorticoid Receptor Pathway

SGK-1, a downstream mediator of Mineral Corticoid receptors (MRs), activates sodium channels (ENaC) which can lead to salt adaptation and eventually hypertension. Sodium intake upregulates SGK-1, thus activating MRs and ultimately activating ENaCs. In normal individuals, Aldosterone elevates SGK-1 levels and activates ENaCs. However, in hypertensive individuals, aldosterone does not elevate SGK-1 levels and decreases ENaCs expression. Therefore, MRs activation by aldosterone, salt, and Rac1 is key in causing salt-sensitive hypertension (Farjah et al., 2003; Pilic et al., 2016).

#### Aldosterone Independent Mineral Corticoid Receptor Pathway (Rac-1)

MRs have also been observed to be activated by Rac-1 (a small GTPase, member of the Rho-guanine triphosphate hydroxylases family). Salt loading increases Rac1 activity in hypertensive individuals. This activates SGK-1 and MRs, which leads to sodium retention in kidneys, and eventually hypertension. Treatment with Rac-1 inhibitors can decrease blood pressure and salt sensitivity (Shibata et al., 2011). Thus, Rac1 is an upstream regulator of MRs and determines BP salt sensitivity (Hirohama and Fujita, 2019).

#### Renin-Angiotensin-Aldosterone System

The renin-angiotensin-aldosterone system (RAAS) is the main coordinator of blood pressure and water-sodium homeostasis in the body. Normally, the RAAS is suppressed when high salt intake is provided, leading to decreased sodium reabsorption and blood pressure. Low-salt intake significantly increases RAAS components, leading to increased circulating renin and angiotensin II (And II) levels and aldosterone production to stimulate sodium reabsorption (Huan et al., 2012; Fujita, 2014). It has been observed that the RAS is either insufficiently suppressed or overly active in salt-sensitive hypertensive individuals (Fujita, 2014) and experimental rodent models (Kobori et al., 2003; Susic et al., 2011). Several studies have also examined the role of the intrarenal renin-angiotensin system (RAS) and found similar results, as evidenced by increased kidney renin and angiotensin II (Bayorh et al., 2005; Susic et al., 2011). For example, high salt feeding for 4 weeks in spontaneously hypertensive rats, failed to suppress RAS activity (Susic et al., 2011). An important feature of RAAS/RAS overactivation in salt sensitivity is the development of renal disease, both preceding and/or as a consequence of hypertension. RAS inhibition ameliorated salt-induced renal injury, indicating that continuous RAS activity contributes to organ damage in salt-sensitive hypertension (Varagic et al., 2008; Susic et al., 2009, 2011). In that regard, chronic Angiotensin II infusion combined with a high salt diet in rats has been shown to significantly increase renal infiltration of immune cells and ROS activity, demonstrating an important immunological role for RAAS in inducing renal injury (Ozawa et al., 2007; Lara et al., 2012; Itani et al., 2016; Norlander et al., 2017).

#### Sympathetic Nervous System Activity

The Sympathetic Nervous System (SNS) is one of the key players that modulate renal function. Increased SNS activity has been observed in salt-sensitive hypertensive patients, especially in the kidneys, causing increased renin secretion, reduced renal blood flow, and increased renal tubular reabsorption (DiBona, 2005; Lohmeier et al., 2012). Under normal conditions, With-no-lysine kinase 4 (WNK4) inhibits the thiazide-sensitive sodium chloride cotransporter (NCC), resulting in increased renal sodium excretion. However, stimulation of adrenergic receptors, modeling the SNS, leads to WNK-4 downregulation and thus activation of NCC. This leads to sodium retention and increased blood pressure. This shows that the SNS can cause salt-sensitive hypertension through the WNK4-NCC pathway (Luzardo et al., 2015).

#### Genetic Mechanisms

Several gene mutations and variants can be related to salt-sensitive hypertension. It is difficult to name all the genes related to salt-sensitive hypertension due to the various pathways and enzymes involved and the different mutations arising in different populations. However, some of the most studied and prominent genes related to salt-sensitive hypertension have been described. BP changes have been associated with DNA methylation in human genome-wide studies. Methylation levels, especially in promoter regions of genes. Pojoga et al. (2011) reported that mice with heterozygous knockout of lysine-specific demethylase-1 (LSD1, Kdm1a) induce histone H3 lysine 4 (H3K4) or H3K9 demethylation, had salt-sensitive hypertension. These mice were also associated with enhanced vascular contraction despite the suppressed RAAS and reduced relaxation via the NO-cGMP pathway. Therefore, a functional role of LSD1 in the development of salt-sensitive hypertension was deduced (Pojoga et al., 2011). In relation to the RAS, HSD11B2 is a gene encoding the kidney (11-HSD2) isozyme of 11β-hydroxysteroid dehydrogenase, which is associated with reduced activity of the protein resulting in salt sensitivity. The activity of this gene was lower in salt-sensitive individuals in comparison to salt-resistant individuals. The genotype of these people was further studied. Agarwal noticed that polymorphism G1065A exposed that the AA + GA genotypes were more frequent in salt-resistant individuals, suggesting a protective role for the A allele (Agarwal et al., 2000; Caprioli et al., 2008). Another gene, the cytochrome P-450 3A5 gene (CYP3A5), and its variants CYP3A5*1 (expressor) and *3 (reduced-expressor) were found related to salt sensitivity. Individuals with *1 carrier had higher blood pressure than *3/*3, but only those with low salt intake, suggesting that CYP3A5 variants could be implicated in salt sensitivity (Zhang et al., 2010).

#### Renal Mechanisms of Salt-Sensitive Hypertension

The role of volume overload and impaired kidney natriuresis in salt-sensitive pathophysiology has been controversial. Some have argued that a certain degree of kidney dysfunction is necessary for the initiation of salt-sensitive hypertension. Moreover, most genetic alterations detected to date suggest that salt sensitivity is principally dependent on renal sodium handling. Defective natriuresis results in positive sodium and fluid balance, which rapidly causes hypervolemia or extracellular fluid volume expansion. Hypervolemia increases cardiac output in an attempt to improve kidney perfusion pressure and sodium and fluid excretion. This is accompanied by compromised kidney autoregulation, which further contributes to increased blood pressure. Data obtained from salt-sensitive individuals (Campese et al., 1991; Bidani et al., 2009; Burke et al., 2014) and experimental models (Karlsen et al., 1997; Burke et al., 2014; Ren et al., 2014) demonstrated dysfunctional renal vascular autoregulation.

In addition to the above, it has been shown that, in response to a declining kidney function, which is commonly seen with hypertension, RAAS activation increases blood pressure, total peripheral resistance, and kidney perfusion pressure to preserve glomerular filtration rate (GFR). In combination with a net loss of overall GFR, Augmented Ang II levels further amplify Na^+^ and fluid retention through stimulation of sodium reabsorption at the proximal tubule and the collecting duct (Ku et al., 2019). This effect is intensified in salt-sensitive individuals (Koomans et al., 1982). Importantly, elevated renal perfusion pressure also increases kidney immune cell infiltration, which leads to further kidney damage (Mori et al., 2008).

### Vascular Dysfunction

As mentioned before, the role of salt loading and impaired kidney natriuresis in the initiation of salt-sensitive hypertension is controversial. Many investigators attribute this pathology to dysfunctional regulation of systemic vascular resistance rather than abnormal increases in renal sodium retention or cardiac output (Sullivan et al., 1987; Greene et al., 1990; Khalil, 2006; Schmidlin et al., 2007, 2011). This theory of vaso-dysfunction holds that in response to salt loading, salt-sensitive individuals do not exhibit increases in cardiac output or sodium retention that are larger than those occurring in their salt-resistant counterparts (Sullivan et al., 1987; Greene et al., 1990; Schmidlin et al., 2007, 2011). Indeed, both salt-resistant and salt-sensitive subjects experience increases in cardiac output, but only salt-resistant individuals can offset the hypertensive effect of increased cardiac output by decreasing systemic vascular resistance. This contrasts with salt-sensitive subjects who demonstrate an inadequate decrease in systemic vascular resistance or a paradoxical increase in vascular resistance that increases mean arterial pressure. This is supported by observations in salt-sensitive individuals that the suboptimal decrease in systemic vascular resistance precedes blood pressure increase, indicating that the abnormal vascular resistance response is not a consequence of salt-induced increases in blood pressure (Morris et al., 2016). Importantly, the abnormal resistance response may vary across organs/tissues (Liard, 1981; van Paassen et al., 1996; Schmidlin et al., 1999). It is possible that this phenomenon is more pronounced at the kidney level and can significantly contribute to blood pressure elevation (van Paassen et al., 1996). Currently, it remains unclear which vasodilatory systems are responsible for this abnormality (Morris et al., 2016). Nitric Oxide (NO) is a potent vasodilator proposed to be a key regulator of vascular tone in salt sensitivity (Bech et al., 1998). In this regard, an important model of salt sensitivity that has been widely used includes a low dose of the nitric oxide synthase inhibitor, L-NAME, that does not elevate blood pressure *per se*, followed by a high salt diet (Itani et al., 2016; Van Beusecum et al., 2019; Wang et al., 2020). This model demonstrated increased blood pressure in response to salt intake due to subnormal vasodilatory responses combined with a normal salt-and salt-induced hypervolemia. Interestingly, controls’ blood volume reductions were associated with elevated urinary sodium excretion, indicating that high blood pressure, sodium retention, and hypervolemia are necessary (Wang et al., 2020).

### High-Fructose Induced Hypertension

Rats fed fructose over a long period gain weight and develop abdominal obesity (Tappy, 2018). In addition to that, feeding the rats with HFD increased the body weight of rats, BMI, AC/TC ratio, and adiposity index compared to the normal control group (Pai et al., 2020). Chronic fructose feeding renders animals leptin resistant. This disrupts normal energy homeostasis, favors positive energy storage, and thus predisposes the animal to obesity (Shapiro et al., 2008). High fructose intake has been connected to increased calorie intake and weight gain, and all of these factors are linked to high blood pressure. Epidemiological, animal and human studies correlate high fructose intake with increased blood pressure. In the International Study of Macro/Micronutrients and Blood Pressure in 2011, data collected from nearly 2,700 individuals presented a direct association of the sugar consumption (glucose, fructose, and sucrose) and sugar-sweetened beverages (SSB) with blood pressure increase independently from body weight and height (Brown et al., 2011). The International Study of Macro/Micronutrients and Blood Pressure also reported the direct correlation between sugar intake and blood pressure in a study on 4,680 men and women aged 40–59 years from Japan, China, the United Kingdom, and the United States (Chan et al., 2016). US adults who consumed added sugar more than 74 grams of fructose per day showed an increase in blood pressure independently from any hypertensive history (Jalal et al., 2010) and that US adults who consumed fewer SSBs per day for 18 months showed a significant reduction in their blood pressure (Chen et al., 2010). Animal studies have shown that dogs fed with a diet containing 60% of the calories as fructose for 4 weeks showed a significant increase in blood pressure, while dogs fed with 60% of the calories dextrose remained normotensive (Martinez et al., 1994). Mice fed with a diet in which 98% of the carbohydrates were from fructose for 7 weeks showed an increase in blood pressure (Farah et al., 2007) while rats fed with a high fructose diet showed increased blood pressure within 3–4 weeks (Juan et al., 1998; Hsieh et al., 2005). A high fructose diet induces hypertension through various mechanisms that interact and crosstalk with each other.

#### Inflammation

Studies showed that high fructose is associated with chronic inflammation. Glushakova et al. (2008) reported that treating human aortic endothelial cells with physiologic concentrations of fructose increases ICAM-1 mRNA and protein expression in a time- and dosage-dependent manner. Furthermore, high fructose increases the systemic secretion of inflammatory cytokines like IL-6 and IL-12 (Porto et al., 2015) and activates the inflammatory signaling in different tissues like the liver, heart, and kidney (Miller and Adeli, 2008). In addition, fructose-induced endothelial dysfunction is associated with significant macrophages and T cells infiltration in perivascular adipose tissue (Jia et al., 2014). It is also reported that TNF-α, IL-1β, and IL-6 are secreted under a high fructose diet, involved in insulin resistance, endothelial dysfunction, and chronic inflammation (Zhang et al., 2017). Further studies are needed to identify the role of immune cells and inflammatory cytokines in fructose-induced hypertension.

#### Endothelial Dysfunction

Endothelial cells play an important role in regulating vascular tone and thus blood pressure by synthesizing and releasing contracting and relaxing molecules. There is evidence that high-fructose diets impair endothelial activities. To illustrate, nitric oxide (NO) has long been recognized as a potent endothelium-released BP-lowering vasodilator generated by endothelial nitric oxide synthase (eNOS). According to Palanisamy and Venkataraman (2013), the expression of eNOS in fructose-fed rats is reduced to half in comparison to that of control rats, thus inducing hypertension. Alternatively, other studies reported that fructose increases the synthetic rate of vasoconstrictors, including Endothelin-1 (ET-1) and Angiotensin II (Ang II). A research observed increased blood pressure and ET-1 expression after feeding Wistar rats 60% fructose diet for 8 weeks (Abdelrahman et al., 2018). These results were expected since Endothelin-1 is a powerful vasoconstrictor that has been linked to an elevation in blood pressure in fructose-fed rats.

Additionally, the role of Ang II in fructose-induced hypertension has been supported by several studies. According to the researchers, endothelial cells expression of angiotensin type I receptors (AT1) was reported to be up-regulated in fructose-fed rats, and treatment with AT1 receptor antagonists reduces the effects of fructose on blood pressure (Shinozaki et al., 2004). Moreover, it was hypothesized that an interrelationship between ET-1 and Ang II may play a role in fructose-induced hypertension. Interestingly, the hypothesis was validated, and results indicate that Ang II depends on the actions of ET-1 to develop fructose-induced hypertension (Tran et al., 2009). Excess dietary fructose can cause endothelial dysfunction in various ways, according to the pathways discussed above.

#### Activation of Renal Sympathetic Nervous System

Research on the role of renal nerves in long-term control of arterial pressure and hypertension implied that renal sympathetic nerve activity (RSNA) plays a critical role in blood pressure regulation (Osborn and Foss, 2017). The mechanisms of elevated RSNA in hypertension pathophysiology have been previously reported, including enhanced renal tubular sodium reabsorption, decreased urinary sodium excretion, decreased renal blood flow and GFR, and increased RAS activity (DiBona, 2005). In favor of this concept, it has been proposed that sympathetic activity rises in fructose enriched environment. An early study reported that sympathectomy (adrenal medullectomy combined with neurotoxic exposure) prevented hypertension in rats fed a high fructose diet (Verma et al., 1999). In line with other studies, Farah et al. (2006) concluded that fructose activates a sympathetic pathway causing an increase in BP in mice. At the molecular level, in fructose-fed mice, responses to alpha-adrenergic blockades were enhanced, indicating an increase in sympathetic nerve activation (Farah et al., 2006). On this basis, the causative effects of fructose on blood pressure increase appear to be the result of complicated and numerous pathways, and the activation of the RSNS is no exception.

### High-Sodium and High-Fructose Induced Hypertension

Existing data indicate a correlation between dietary salt and blood pressure increase. However, the exact mechanism of high salt-induced hypertension is not entirely understood. It is known that there is a difference in salt sensitivity between individuals. Excessive salt intake has been associated with hypertension via mechanisms that are poorly understood. Traditionally, sodium was thought to distribute according to a two-compartment model, in which extracellular sodium was thought to equilibrate between the plasma and the interstitium and promote inflammation (Machnik et al., 2010). Genetics plays a crucial role in salt sensitivity, 74% in blacks (Svetkey et al., 1996) and 50% in the Chinese population (Machnik et al., 2009). Epigenetic factors play a role in salt sensitivity, including obesity, aging, and stress. A Cross-sectional study conducted on 1,688 participants (4–18 years) showed that sodium intake is associated with an increase in sugar-sweetened soft drinks consumption. Reducing salt-intake confines high sugar drinks consumption and decrease the risks of obesity and hypertension (He et al., 2008). Even though fructose intake increases blood pressure in rats (Nakagawa et al., 2006) and humans (Perez-Pozo et al., 2010), researchers reported a little increase in blood pressure with no induction of hypertension as a response to fructose intake (D’Angelo et al., 2005). In this context, salt was concluded to have a permissive effect for fructose-induced hypertension, in which the combination of high fructose (HF) and high salt (HS) was required for a hypertensive response which did not develop in rats fed either alone (Cabral et al., 2014; Klein and Kiat, 2015). This questions the relation between dietary fructose and dietary salt in elevating blood pressure.

#### Increased Salt Absorption

Research has provided evidence for the role of Glut5 in hypertension pathology. Fructose is absorbed on the apical side of enterocytes in the intestine via the Glut5 transporter. While Glut_5_^+/+^ mice enhanced salt absorption in response to a fructose-rich diet for 14 weeks and augmented hypertension, Glut_5_^–/–^ mice did not exhibit fructose-enhanced salt absorption and developed hypotension and nutrient malabsorption (Barone et al., 2009). Salt absorption in the intestine occurs via the sodium transporter Na^+^/H^+^: sodium-hydrogen exchanger 3 (NHE_3_) and the chloride exchangers Cl*^–^*/HCO_3_*^–^*: putative anion transporter 1 (PAT_1_) located at the apical side in the upper small intestine and the proximal kidney tubule and down-regulated in adenoma (DRA), located at the apical membranes in the mid to lower small intestine and the upper large intestine. Dietary fructose increases mRNA and protein levels of NHE_3_ and PAT_1_ in the jejunum. It has been shown in wild-type mice that increased dietary fructose intake increased the expression of PAT_1_ and Glut_5_ and increased salt absorption, while PAT_1_ knock-out mice showed no increase in salt absorption (Singh et al., 2008). Similarly, fructose-induced salt absorption was abolished in NHE_3_ (+ / +) mice (Soleimani and Alborzi, 2011). Taken together, these studies and others present a critical role of Glut5, NHE_3_, and PAT_1_ in fructose-induced salt absorption.

#### Decreased Sodium Excretion

Excess dietary fructose activates the RAS in the kidneys and enhances the proximal tubular sodium reabsorption. For example, a week of fructose (20%) feeding did not change the rat’s blood pressure while adding high salt to diet-induced hypertension. Fructose stimulated NHE_3_ activity in proximal renal tubules and sensitized the rats to angiotensin II (Ang II) (Cabral et al., 2014). Molecularly, diacylglycerol (DAG), a fructose metabolite, activates protein kinase C (PKC), which in its turn sensitizes the proximal tubules to Ang II. Others have shown that fructose overload upregulates the Na^+^/K^+^-ATPase expression in the kidney (Rukavina Mikusic et al., 2018).

Two weeks of fructose (20%) feeding has been shown to upregulate the renal expression of the pro-renin-receptors (PRR) abrogated by allopurinol. Similarly, soluble PRR protein expression increased in response to High Fructose and uric acid (UA) in cultured HK-2 cells in a dose- and time-dependent manner. High Fructose also increased the renal NHE_3_ and Na^+^/K/2Cl cotransporter (NKCC_2_) expression and *in vivo* NKCC2 activity, which was abolished by a PRR inhibitor PRO20 or allopurinol. High Fructose also leads to activation of the intrarenal RAS system (Xu et al., 2017). This data together supports the role of fructose, via its metabolite uric acid, increases Na^+^ reuptake by upregulating PRR, which stimulates intrarenal RAS regulates NHE_3_ and NKCC2 expression. Moreover, fructose promotes sodium absorption by enhancing glucocorticoid activity with an anti-natriuretic effect. Rats drinking 10% fructose solution had increased expression level of 11beta-hydroxysteroid dehydrogenase Type 1 (11βHSD1) and hexose-6-phosphate dehydrogenase (H6PDH) and adipose tissue, together with enhanced GR nuclear accumulation (Bursać et al., 2013).

#### Fructose-Salt-Insulin Interaction

Several mechanisms have been proposed to explain the pro-hypertensive nature of Insulin, including activation of the sympathetic system (Rowe et al., 1981), growth-promoting activity on vascular smooth muscle cells (Pfeifle and Ditschuneit, 1981), and increased renal sodium reabsorption. Several studies have been performed in rats, dogs, and humans to delineate the anti-natriuretic effect of Insulin. Given that Insulin increases Na^+^ reabsorption by directly affecting the renal tubules through the epithelial Na^+^ channel (ENaC) (Zhang et al., 2005), to investigate the fructose-insulin-salt interaction, Sprague-Dawley rats 3 weeks of high fructose diet (66%) with either low (0.07%), normal (0.3%), or high (7.5%) NaCl concentrations. High fructose feeding with normal or high salt increased rat blood pressure, yet not with low salt. A decrease in the insulin receptor and mRNA expression in tissues was observed in response to salt alone as a protective mechanism to prevent excessive Na^+^ reabsorption in the presence of the high salt diet. Nonetheless, when fructose is introduced into the diet, high salt fails to reduce insulin receptor or mRNA levels in the kidneys, causing an increase in salt reabsorption and decreased urine Na^+^ concentration (Catena et al., 2003). The regulation of ENaC by Insulin involves the serum and glucocorticoid-inducible kinase (SGK_1_) through the activation of Phosphatidylinositide-3 (PI_3_)-kinase (Park et al., 1999) and phosphoinositide-dependent kinase 1 (PDK_1_) (Biondi et al., 2001). Rats that lack SGK1^–/–^ failed to establish hypertension in response to high salt/high fructose (HS/HF) diet (Huang et al., 2006). On the other hand, α-Lipoic acid improves insulin sensitivity and prevents hypertension in rats fed a high fructose diet for 20 days (Thirunavukkarasu et al., 2004).

#### Endogenous Fructose Production

High salt diets have been linked to an increased risk of diabetes, leptin resistance, and obesity, regardless of calories (Lanaspa et al., 2018). The relationship between salt and fructose may be more complex; according to recent research, a high-salt diet may stimulate systems in the liver that result in fructose synthesis (endogenous fructose production) (Lanaspa et al., 2018). HS dietary intake activates the liver aldose reductase-fructokinase pathway leading to the production of fructose. In mice, it has been shown that HS intake stimulates Tonicity-responsive enhancer-binding protein (TonEBP or NFAT5), a regulator of cellular adaptation to hypertonicity and inflammation like macrophage activation and T-cell development (Lee et al., 2019). TonEBP activates the aldolase reductase, which induces the conversion to sorbitol, further metabolized to fructose by the sorbitol dehydrogenase. Fructose metabolism leads to ATP depletion, leptin resistance, fatty liver diseases, and metabolic syndrome with hypertension. Another example of the simultaneous role of fructose and salt in developing hypertension was when the fructose metabolism was blocked in fructokinase knockout mice, preventing the effects induced by HS (Lanaspa et al., 2018).

#### Oxidative Stress

It has been shown that high fructose induces ROS levels, thus contributing to elevated blood pressure. Excessive oxidative stress levels arise with HS/HF diet. Studies have shown that feeding rats a 40% fructose and 3% NaCl-rich diet significantly decreased eNOS in the renal medulla compared to the rats fed a high salt diet alone (Nishimoto et al., 2002). Not only did blood pressure increase significantly in the HF/HS group (20% fructose (supplemented in drinking water); 4% NaCl) compared to the HF and the HS groups, but also the sodium excretion and the Nitric oxide (NO) excretion measured with the levels of NO metabolites (NO_2_/NO_3_) were significantly reduced in the HF/HS group (Gordish et al., 2017). Thus, Reduced NO availability contributes to fructose-induced salt-sensitive hypertension. According to Hall et al. (2012) nitric oxide deficiency causes salt-sensitive hypertension via several mechanisms, including improved sensitivity to vasoconstrictors, increased renal tubular sodium reabsorption, increased renal vascular resistance, and amplified renin release. Further investigation is warranted to identify the mechanisms by which salt and fructose promote ROS production.

### Sodium and Fructose Intake Levels in Lebanon

**FIGURE 1:**
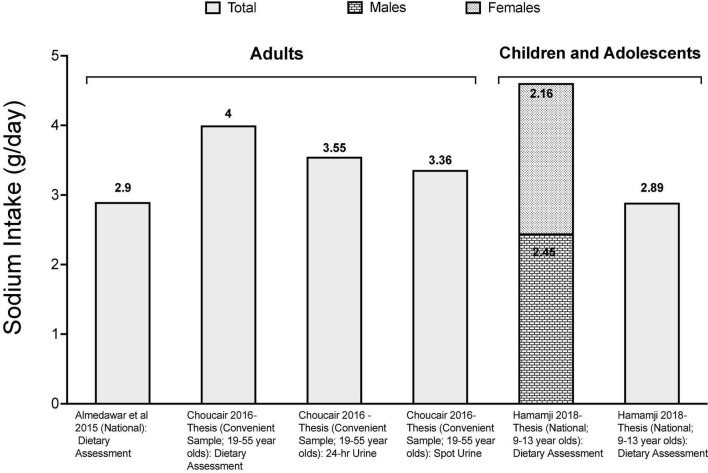
Available data on sodium intake levels in Lebanon. References: *For adults:* Almedawar et al. (2015) and Chouccair (2016)-Thesis. *For children and adolescents:* Merhi (2017)-Thesis and Hamamji (2018)-Thesis.

Several studies have reported high salt intakes in Lebanon (Figure 1). Based on Bayesian hierarchical modeling, Powles et al. (2013), reported a sodium intake estimate of 3.130 g/person/day in Lebanon, and this intake level was estimated to contribute to 210 cardiometabolic deaths per million adults (Afshin et al., 2015). The estimate reported by Powles et al. (2013), is in line with values reported by dietary assessment studies conducted in Lebanon, showing that salt intake among adults is higher than the recommended maximum intake level. For instance, using data stemming from a nationally representative survey conducted in 2008/2009 amongst Lebanese adults (*n* = 2,543), average sodium intake was estimated at 2.900 g of sodium, with up to 60% of individuals consuming more than the upper limit of 2.000 g/day (Almedawar et al., 2015). Other dietary studies conducted amongst adults have reported even higher estimates ranging between 3.390 g/day (± 1.283) and 4.618 g/day (± 1.898) (Chouccair, 2016). The primary food groups contributing to daily salt intake were identified and included bread and bread-like products (26% of sodium intake), processed meat (12%) and dairy products (9%) (Almedawar et al., 2015). Studies based on 24-h urine collection, which is considered as the gold standard for the assessment of salt intake, reported an average intake of 3.550 g/day (± 1.654) while estimates based on spot urine analysis provided an estimate of 3.357 g/day (± 0.860) (Chouccair, 2016). Although different studies may use different methods of sodium intake assessment, intake levels observed amongst Lebanese adults can be compared to those reported by other countries. Accordingly, sodium intakes in Lebanon were found to be higher than those reported from Austria (2.2 g/day) (Hasenegger et al., 2018) and the UAE (2.71 g/day) (Jarrar et al., 2020), while being lower than estimates reported from Japan (4.16 g/day) (Udagawa et al., 2008), England (3.3 g/day) (Public Health England, 2020), Oman (4.12 g/day) (Ministry of Health-Oman, 2017; Al-Mawali et al., 2021), the US (3.47 g/day) (Clarke et al., 2021), Egypt (3.5 g/day) (Central Agency for Public Mobilization and Statistics et al., 2018), Iran (3.74 g/day) (Ministry of Health and Medical Education et al., 2016; Rezaei et al., 2018), Morocco (4.17 g/day) (The Kingdom of Morocco and World Health Organization, 2018) and China (5.01 g/day) (Fang et al., 2020).

Studies conducted amongst children and adolescents in Lebanon have also reported high sodium intake. In a national study of children aged 6–10 years, the average sodium intake was estimated at 2.894 ± 0.726 g/day based on spot urine analysis (Merhi, 2017); while dietary studies reported values ranging between 2.160 g/day (± 0.800) in 9–13-year-old girls and 2.450 g/day (± 800) in boys (Hamamji, 2018).

**FIGURE 2:**
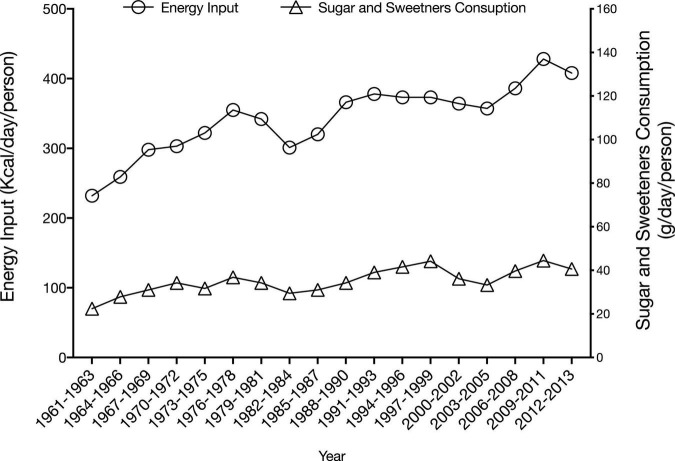
Trend in dietary supply of sugar and sweeteners in Lebanon (Food and Agriculture Organization of the United Nations, 2021).

Data on sugar intake is scarce in Lebanon. Based on food availability data that are published by FAOSTAT (Food and Agriculture Organization of the United Nations, 2021), sugar supply was found to increase from 70 g (contributing 232 kcal) per person per day in 1961-63 to 127 g (408 kcal) in 2012–2013 (Figure 2). In a study conducted amongst Lebanese adults living in the capital Beirut, dietary fructose intake was estimated at 51.4 g/day, an estimate that is similar to that reported from the US (48.07 ± 35.73 g/day), while exceeding values reported from European countries, such as Germany and Finland, where fructose intake ranged between 18.4 and 40.6 g/day (Montonen et al., 2007; Schulze et al., 2008). This estimate of dietary fructose intake in Lebanon was also found to exceed the suggested upper limit of fructose intake (50 g/day) (Johnson et al., 2009).

Based on the WHO definition of free sugar (FS), which includes added sugar in addition to sugars naturally present in honey, syrups, fruit juices, and fruit juice concentrate, a recent study estimated free sugar intake amongst Lebanese children and adolescents based on two national surveys. The study showed that the contribution of FS to daily energy intake (EI) increased from 8.5% in under-five children to 11.9% in school-aged children and adolescents (aged 5–18 years) (Jomaa et al., 2021). More than 40% of under-five children were found to exceed the WHO upper limit for FS intake (10% EI), while this proportion increased to 62% in older children and adolescents (Jomaa et al., 2021). The top contributors to FS intake in both age groups (under-five children and 5–18 years old) were SSBs and biscuits and chocolates (Jomaa et al., 2021).

Other available data suggest a high intake of sweetened foods and beverages in Lebanon. Based on a national survey, the consumption of sweets and SSB was reported to be in the range of 18% of energy intake in adults (Nasreddine et al., 2020). In a study that aimed at assessing the contribution of various dietary risk factors to cardiometabolic deaths in the EMR, Afshin et al. (2015) estimated the intake of SSBs at 185 g/day in Lebanon, an intake level that was associated with 59 cardiometabolic deaths per million adults in the country. More alarming is the observed trend in SSB consumption in Lebanon. A recent national study documented an increase in SSB among Lebanese adolescents, from 241.4 g/day in 1997 to 321.7 g/day in 2009 and 301.6 g/day in 2015 (Naja et al., 2021).

Like other countries of the EMR, Lebanon has witnessed a westernization of dietary practices during the past two decades, losing part of its cultural food heritage and dietary idiosyncrasies (Sibai et al., 2010; Nasreddine et al., 2019a). These shifts in diet and food consumption habits have been particularly seen as of the 1990s, after the end of the Lebanese civil war (Nasreddine et al., 2019a), when Lebanon experienced drastic socio-economic and socio-cultural transformations that have led to important changes in the food supply chain and food demand. Examples of such changes include rapid urbanization, technical development in the food industry sector, and the increasing importation of foods. These transformations may partially explain the observed changes in dietary patterns amongst the population, with lower adherence to the traditional Lebanese dietary pattern and a wider embracement of the western dietary patterns, characterized by higher salt and sugar contents (Sibai et al., 2010). These facts underline a public health concern, given that the western dietary pattern has been consistently associated with a higher risk of obesity, metabolic syndrome, and chronic disease.

### Salt and Sugar Intake Reduction Strategies in Lebanon

Efforts to reduce salt and sugar intake are rather timid in Lebanon. National strategies and action plans for salt or sugar intake reduction are lacking (Al-Jawaldeh et al., 2021), and there has been no establishment of national targets for the population intake levels (Al-Jawaldeh et al., 2021). This is of concern given that governmental commitment has been described as an essential prerequisite for nutrition policy development and implementation to facilitate the availability and consumption of safe and nutritious foods that make up a healthy diet, including low salt and low sugar diets (Mozaffarian et al., 2018). Despite the lack of national agendas or strategies for reducing salt, the Lebanon Action for Salt and Health (LASH) initiative was launched in February 2012 as part of the Vascular Medicine Program at the (American University of Beirut Medical Center [AUBMC], 2014). The Lebanese Action for Sodium and Health (LASH) is one of 29 worldwide collaborating countries and members of the World Action of Salt and Health (WASH), a World Health Organization (WHO) initiative to raise salt awareness (American University of Beirut Medical Center [AUBMC], 2014). The main objectives of LASH are to optimize salt intake among the Lebanese population by raising awareness on salt-related health risks, providing professional training, conducting original salt research, connecting with global salt optimization leaders, and advocating for the development of salt reduction policies. LASH has been active in generating data on the population salt intake levels (Almedawar et al., 2015), salt levels in food products (particularly bread as the main staple food in Lebanon) (Food and Agriculture Organization of the United Nations, 2007), and in assessing the consumer’s knowledge, attitude, and behavior (KAB) toward salt (Nasreddine et al., 2014).

The research on KAB has highlighted important gaps in consumers’ knowledge, such as the relationship between salt and health (Nasreddine et al., 2014). In a study conducted amongst Lebanese consumers visiting supermarkets in Lebanon, less than one-quarter of the study population (21.5%) had correctly identified the main contributors of salt in the Lebanese diet, while more than a third overlooked salt content data on the food labels (Nasreddine et al., 2014). These findings will be used to develop culture-specific interventions to raise awareness amongst Lebanese consumers.

On another front, LASH contributes to developing product reformulation policies and establishing a national salt monitoring and evaluation system. LASH has collaborated with the Ministry of Industry and the Ministry of Health in Lebanon to voluntarily develop national salt standards in bread (American University of Beirut Medical Center [AUBMC], 2014; World Health Organization Regional Office for the Eastern Mediterranean, 2015). Legislation on salt reduction should have been proposed to concerned members of Parliament (World Health Organization Global Database on the Implementation of Nutrition Action [GINA], 2014). The scope of work of LASH will also include the establishment of a Lebanese salt-sensitive cohort in normotensive, pre-hypertensive, and hypertensive patients to identify inflammatory markers, genetic, and microbiome changes, improving health literacy within the Lebanese community with regards to high blood pressure and CVD; designing educational courses and flyers focusing on salt reduction, and developing and launching a “salt awareness campaign nationwide in association with the Lebanese Society of Cardiology (LSC) and Vascular Medicine Program.

Efforts on sugar reduction are lacking in Lebanon, and policies focusing on sugar reduction are completely absent. In addition, only a few studies have been conducted to assess sugar intake in the population (Afshin et al., 2015; Nasreddine et al., 2020; Jomaa et al., 2021; Naja et al., 2021), or to investigate the food environment in aspects pertinent to sugar consumption. For instance, acknowledging that exposure to food marketing may influence children’s food preferences and consumption patterns, including their consumption of sugar-sweetened foods and beverages, a recent study has performed a content analysis of food advertisements on local TV channels during children’s viewing time in Lebanon (Nasreddine et al., 2019b). The results showed that approximately 8 out of 10 food advertisements were for products that did not meet the standards specified by the WHO nutrient profile model and which sets specific criteria for foods/beverages that can be marketed to children based on their sugar, salt, and fat content. The study also showed that chocolates and sugar confectionaries and cakes and sweets were the most frequently advertised food categories to children, while savory snacks, cheeses, and sweetened beverages were also among the commonly advertised food categories. These findings suggest that television food advertising during children’s viewing times disproportionately promotes consuming foods high in fat, sugar, and salt, thus promoting unhealthy dietary patterns in children (Nasreddine et al., 2019b). Finally, food-based dietary guidelines (FBDGs) have been developed in Lebanon and include spelled recommendations pertinent to salt and sugar intake reduction (American University of Beirut, 2013). However, wider dissemination of these guidelines is needed to enhance its impact on dietary habits.

### Recommendations for Policy Development and Future Research

There is a dire need to develop and implement population sodium and sugar reduction policies in Lebanon. Securing governmental commitment toward these goals and establishing national salt and sugar reduction targets are needed to develop and implement such policies. More importantly, these policies and interventions ought to be multifaceted in their approach, including (1) product reformulation through engagement with the food industry in order to progressively reduce sodium/salt and sugar in processed food products; (2) implementation of consumer education and awareness campaigns to raise consumer awareness in relation to healthy dietary practices and the need to reduce salt and sugar intake; (3) the establishment and adoption of consumer-friendly food nutrition labeling regulations that include salt and sugar; (4) interventions within specific settings such as schools, workplaces, public institutions (World Health Organization, 2016); (5) implementing fiscal measures to encourage the production and consumption of foods with reduced salt and sugar content and (6) limiting the marketing of foods and beverages that are high in sugar and salt, as per the WHO recommendations (World Health Organization, 2016).

The implementation of multifaceted interventions is expected to be significantly more impactful as compared to fragmented and unidirectional interventions. For instance, the potential impact of 3 policies to decrease dietary salt intake was assessed in some countries of the EMR: a health awareness campaign, the labeling of packaged food, and the mandatory reformulation of processed foods to decrease salt content (Mason et al., 2014). The cumulative population health effects were determined as life-years gained (LYG) over a 10-year time frame. Findings showed that for Palestine, the combination of the three policies mentioned above, which would result in a 30% reduction in salt intake, would lead to an estimated cost savings of $6,000,000 and 2,682 life-years gains. In Syria, the combination of these three policies would result in an estimated cost savings of $39,000,000, and 31,674 life-years gained (Mason et al., 2014), while in Tunisia, this would be yield an estimated cost savings of $235,000,000 and 6,455 life-years gained (Mason et al., 2014). It is also important to highlight that incorporating a legislative component within these strategies and policies would be more impactful than implementing solely voluntary initiatives. Previous systematic reviews and modeling studies have emphasized that mandatory or legislative approaches tend to be more effective, producing larger reductions in the population’s salt or sugar intake levels (Nghiem et al., 2015; Hope et al., 2017; Hyseni et al., 2017). Implementing clear monitoring approaches is also essential to demonstrate program effectiveness and incite greater changes in salt and sugar intakes (Webster et al., 2014).

There is also the need for additional research to support policy development and implementation. Future studies should assess salt intake in various population and age groups, using validated methods such as the 24-h urinary excretion; evaluate sugar intake in various segments of the population using validated dietary approaches, and assess the impact of salt or sugar reduction on the organoleptic properties of culture-specific staple foods such as bread. Research should also examine the acceptability and effectiveness of different front-of-pack labeling schemes in Lebanon to support adopting such schemes in the country. Translational research projects in inflammation-mediated cardiovascular and renal disease are also needed, particularly salt-sensitive hypertension and oxidative stress changes leading to activation of the immune system. Similarly, it is crucial to design patient-targeted assessment tools, including cardiovascular health risk factors and health literacy.

## Conclusion

**FIGURE 3:**
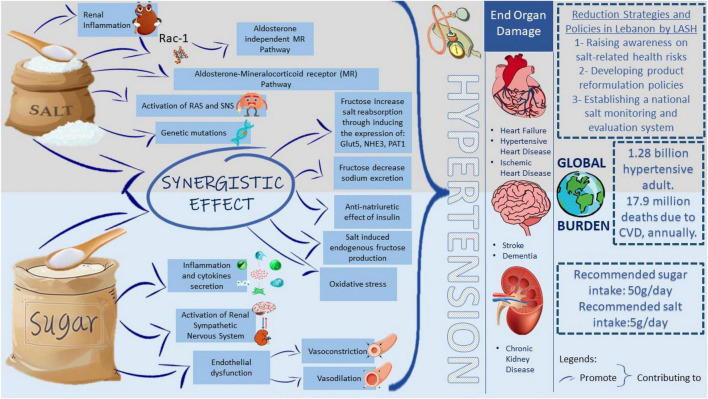
Proposed mechanisms by which salt and fructose consumptions lead to the hypertensive global burden. The high incidence of hypertension imposes a significant public health burden as 1.28 billion adults are hypertensive worldwide. Having hypertension increases the risk of having CVD, stroke, or chronic kidney disease. The mission of the LASH initiative is to optimize salt intake among the Lebanese population by imposing different reduction strategies and policies which aid in reducing the health burden from elevated blood pressure. The upper panel includes different pathways triggered by salt intake, which increases blood pressure. The lower panel includes different pathways triggered by sugar intake, which increases blood pressure. In the middle, the synergistic effect of salt and sugar on elevating blood pressure. WHO recommends 50 g of sugar intake and 5 g of salt intake per day. Rac-1, Rac family small GTPase 1; MR, mineralocorticoid receptor; RAS, renin-angiotensin system; SNS, sympathetic nervous system Glut5, glucose transporter 5; NHE3, sodium/hydrogen exchanger 3; PAT1, putative anion transporter 1; CVD, cardiovascular disease; LASH, Lebanese Action on Salt and Health.

This review highlights how high fructose and salt intake may contribute to the increasing burden of hypertension, a cardiovascular condition threatening population health and development (Figure 3). Salt has multiple effects on the immune system, causing nephritis, sodium reabsorption, and thus hypertension. Other mechanisms by which salt induces hypertension include the activation of MRs, RAS, and SNS. Besides, different genetic mutations and variants have been related to salt-sensitive hypertension. Similarly, fructose may induce intestinal, renal, and endothelial abnormalities and eventually lead to hypertension, insulin resistance, adipogenesis, and non-alcoholic fatty liver disease (NAFLD).

Interestingly, different studies claim a synergistic relation between high salt and high fructose diets in inducing hypertension. For example, fructose has increased intestinal salt absorption by expressing specific transporters and decreasing sodium excretion by activating the RAS. In addition, some studies showed that fructose induces insulin expression, which has an anti-natriuretic and thus exerts a hypertensive effect. Available evidence suggests that the synergistic relation between salt and sugar may be bidirectional. Understanding the co-dependence of salt and fructose in regulating blood pressure may pave the way for new hypertension therapy approaches that focus on the various contributory pathways identified. Even though salt reduction was one of the top 3 priorities to reduce premature mortality from NCD by 25% by 2025 (Beaglehole et al., 2011), salt intake remains high in Lebanon. LASH has been working on raising salt awareness and optimizing salt consumption in Lebanon, yet the efforts on sugar reduction in the country are still completely absent. In an effort to curb the CVD epidemic in the country, there is a dire need for public health strategies aimed at reducing the intake of salt and sugar at the population level.

## Author Contributions

ML, MI, DM, ZR, LN, and HI contributed to the writing of the manuscript. All authors contributed to the article and approved the submitted version.

## Conflict of Interest

The authors declare that the research was conducted in the absence of any commercial or financial relationships that could be construed as a potential conflict of interest.

## Publisher’s Note

All claims expressed in this article are solely those of the authors and do not necessarily represent those of their affiliated organizations, or those of the publisher, the editors and the reviewers. Any product that may be evaluated in this article, or claim that may be made by its manufacturer, is not guaranteed or endorsed by the publisher.
