# Corrigendum: The Sweet and Salty Dietary Face of Hypertension and Cardiovascular Disease in Lebanon

**DOI:** 10.3389/fphys.2022.914438

**Published:** 2022-07-08

**Authors:** Mohammad Labban, Maha M. Itani, Dina Maaliki, Zeina Radwan, Lara Nasreddine, Hana A. Itani

**Affiliations:** ^1^ Faculty of Medicine, University of Balamand, Beirut, Lebanon; ^2^ Department of Pharmacology and Toxicology, Faculty of Medicine, American University of Beirut, Beirut, Lebanon; ^3^ Department of Anatomy, Cell Biology and Physiological Sciences, Faculty of Medicine, American University of Beirut, Beirut, Lebanon; ^4^ Vascular Medicine Program, American University of Beirut Medical Center, Beirut, Lebanon; ^5^ Department of Nutrition and Food Sciences, Faculty of Agricultural and Food Sciences, American University of Beirut, Beirut, Lebanon; ^6^ Adjunct Clinical Pharmacology, Vanderbilt University Medical Center, Nashville, TN, United States

**Keywords:** lifestyle, diet, salt, fructose, hypertension, immunity

“Zeina Radwan” was not included as an author in the published article. The corrected **Author Contributions** Statement appears below:

“ML, MI, DM, ZR, LN, and HI contributed to the writing of the manuscript. All authors contributed to the article and approved the submitted version.”

Furthermore, **Affiliation 1** was listed incorrectly as Balamand. This has been corrected as the “University of Balamand”.

The authors apologize for these errors and state that this does not change the scientific conclusions of the article in any way. The original article has been updated.

